# Reviews

**Published:** 1908-08-15

**Authors:** 


### REVIEWS.

Our Baby. By Mrs. J. L. Hewer. (Bristol : J. Wright and Company. Pp. 168. Eleventh edition. Price 1s. 6d.)

We have read through this popular account of the way to take care of a baby, and we are not surprised to see that its circulation is nearing the sixtieth thousand. From the standpoint of the physician it is sound and practical, and from that of preventive medicine also it is a valuable help; our only shadow of doubt is whether in places it is not a little too technical for those whom it is intended to instruct. Any practising medical man who wishes to recommend a mother, prospective or actual, a book on the proper care of children, will not be disappointed with this publication.

Points of Practice in Maladies of the Heart. By Sir James Sawyer, Consulting Physician to the Queen's Hospital, Birmingham. Pp. 96.

Sir James Sawyer's Lumleian lectures at the Royal College of Physicians are here published in book form. That these lectures are scholarly and interesting goes without saying, but they do not pretend to be anything more than a survey, largely historical, of the past and present attitude of physicians towards cardiac maladies. Perhaps the most noteworthy section is that in which are advocated the merits of a solid wooden stethoscope with a hemispherical chest-piece. We do not gather at what price the volume is issued.

The Radio-active Substances. By Walter Makower. The International Scientific Series, Vol. XCII. (Paul, Trench, Trübner and Company. Pp. 301. Price 5s. net.)

There is little reason to suppose that the present state of knowledge regarding radio-activity and its effect on life or on disease is anything more than a fraction of what it will be a few years hence. Before anyone can investigate the action of any agent upon morbid processes he must be acquainted with the latest researches about that agent; a workman, in fact, must know both the capacity and the limitations of his tools. In this book is set out what is now clearly established about radio-activity, and the physician who thinks of giving his attention to radio-therapy will do well first to read what Mr. Makower has to say on the radio-active substances; he will find it lucid, interesting, and instructive.

Right-handedness and Left-handedness. By G. M. Gould, M.D. Pp. 210. (J. B. Lippincott Company, Philadelphia and London 1908.)

Collected papers setting out the theory that right-handedness is a result of "right-eyedness," since with the majority the right is the dominant or master-eye in fixing objects. It sounds as though there were a good deal in Dr. Gould's notion, in spite of his incompletely proved deductions and his dreadfully faulty style. Thus, "The *tabula rasa* of the infant mind is by no means blank, but its inheritances are necessarily abstract, and are vivified and definitised by the daily millionfold personal—*i.e.*, chiefly retinal—images poured among the ancestral carbon strata awaiting the touch of reality to awaken them to living light and heat." Too many American writers treat us to this sort of composition.

A Second Study of the Statistics of Pulmonary Tuberculosis : Marital Infection. By the late E. G. Pope, M.D., Adirondack Sanitarium. Edited by Karl Pearson, F.R.S., with an Appendix by E. M. Elderton, Galton Research Scholar, University of London. (London : Dulau and Company. 1908.)

Tuberculosis occurring in both husband and wife may be due first (and most obviously) to marital infection; next, in some cases, merely to chance incidence—the disease in question being such a common one; lastly, what has been called "assortative mating" may be made responsible—more colloquially, the principle of "like will to like." Tuberculous proclivities have long been associated with indefinite mental and physical traits, some of which may have considerable sexual attractive power; it is thus possible that male and female candidates for tuberculosis may to some extent select each other as their matrimonial partners to the exclusion of the more healthily disposed. This memoir, based on the author's observations supplemented by records taken from 35 papers cited from literature, finds by advanced mathematical methods that the two last factors have more influence than is usually supposed. The subject is a very difficult and complex one, but it is interesting to find exact statistical confirmation of what some writers of Morel's and of Lombroso's way of thinking have previously asserted—namely, mutually selective mating on the part of abnormal individuals.

